# Social pathways to care: how community-based network ties shape the health care response of individuals with mental health problems

**DOI:** 10.1007/s00127-023-02476-2

**Published:** 2023-04-19

**Authors:** Harold D. Green, Bernice A. Pescosolido

**Affiliations:** 1 Department of Applied Health Science, School of Public Health and the Irsay Institute, Indiana University, Bloomington, IN USA; 2 Department of Sociology, College of Arts & Sciences and the Irsay Institute, Indiana University, Bloomington, IN USA

**Keywords:** Utilization, Network episode model, Social networks, Health disparities, Mental health services

## Abstract

**Purpose:**

Mental health research has powerfully documented inequities related to characteristics, such as ethnicity and gender. Yet how and where disparities like unmet need occur have been more elusive. Drawing from a now modest body of research that deployed the Network Episode Model (NEM), we examine how individuals create patterns of response to mental health problems, influenced by the culture and resources embedded in their social networks.

**Methods:**

The Person-to-Person Health Interview Study (P2P; *N* ~ 2,700, 2018–2021) provides representative, community-based, NEM-tailored data. Both descriptive, latent class and multinomial regression analyses mark mental health care-seeking patterns, including individuals consulted and activities used, as well as the influence of the structure and cultural content of social networks.

**Results:**

Latent class analysis detected five pathways with good fit statistics. The Networked General Care Path (37.0%) and The Kin General Care Path (14.5%) differ only in whether friends are activated in using the general care sector. The Networked Multi-Sector Care Path (32.5%) and The Saturated Path (12.6%) involve family, friends, and both general and specialty care with only the latter expanding consultation to coworkers and clergy. The Null Path (3.3%), or no contacts, is not used as perceived problem severity increases. Network size and strength are associated with the more complex pathways that activate ties, respectively. Trust in doctors is associated with pathways that include specialty providers but not others at work or church. Race, age, and rural residence have specific pathway effects, while gender has no significant impact.

**Conclusions:**

Social networks propel individuals with mental health problems into action. Tie strength and trust produce care responses that are fuller and more targeted. Considering the nature of homophily, results also suggest that majority status and college education are clearly implicated in networked pathways. Overall, findings support community-targeted rather than individually based efforts to increase service use.

## Introduction

The onset of mental health problems presents individuals and society with three unique dilemmas. First, with no physical manifestations, great heterogeneity, few symptoms distinct from behavioral or biological repertories, and no measurable signs to bring to diagnosis, recognizing the problem is daunting for individuals and mental health providers alike [1]. Second, the persistence of public, personal, and provider-based stigma surrounding mental health [2]; the difficulties that individuals from diverse cultures or identities groups face in care [3]; and the documented fear of reprisal that medical providers, themselves, report in facing their own mental health problems [4] all diminish the public’s enthusiasm for seeking formal mental health care. Third, even if individuals want such care, the perennial mental health workforce shortage [5]; efforts to continually pass additional legislation to enforce parity efforts and equitable reimbursement for sustained mental health care (e.g., proposed H.R.1364—Parity Enforcement Act of 2021; see [6, 7]); and the diversion of the majority of U.S. mental health research funds from services, stigma and treatment innovation to a failed search for biomarkers [8] makes the search for care difficult. In essence, the science, the public, and the system all conspire to produce high levels of unmet need. Countering this discouraging profile are the facts that many treatments work [9]; mental health literacy in the public has improved [10]; frameworks, research and interventions to understand and reduce mental health disparities have grown [11–13]; legislation to prioritize physician mental health has passed [4]; and anti-stigma efforts have documented modest but real effects [14–16].

This summary of negative and positive conditions in the mental health landscape reveals a great deal of complexity and uncertainty surrounding the response to onset, care, and outcome. Yet, for the most part, the traditional approach to understanding who eventually gets into care focuses on individual factors and a “one-off event” [17: 140]. Individuals’ assessment and beliefs, their knowledge of insurance and access, and their social characteristics are the mainstay of research [18]. More recently, however, theoretical efforts have expanded to consider how these critical aspects are embedded in communities, both public and professional, where social interactions are the mechanism through which problems are recognized and services are provided. With the response to onset seen as a process that is often “peopled”, the Network Episode Model (NEM) [19, 20] represents one effort. Developed in direct response to the dominant individually focused, use-no use models, the NEM contextualized illness response by suggesting two key theoretical differences. First, tracing the entire set of actions, from discussion with family and friends to consultation with lay advisors to entering the healthcare system, shifts the focus to patterns or pathways to care. Second, by emphasizing that recognizing health problems, especially mental health problems, is difficult even for the educated, the role of social network ties become more central. Individuals in family, work, school, or other social settings hold (mis)knowledge, beliefs, and resources that can facilitate or delay entry into formal mental health care. Further, social network influence likely varies by the structure of ties (e.g., number and frequency), which provide the degree or “push” or influence, in combination with cultural scripts that suggest whether formal mental health care systems can be trusted.

Research employing the NEM has documented different patterns and pathways to care, diverse mental health care experiences (even for anxiety, depression, and mania/hypomania, the most common mental health concerns) and varying outcomes [21–25]. For example, among older individuals, coercion into mental health care was rare, while “muddling through” predominated [22]. Among African American youth, access to mental health services has been shown to be a relational and socially embedded process [26]. Different pathways were associated with diverse outcomes, including diagnosis, trusting working alliances, completing treatment protocols, or post-treatment adherence [21, 27–30]. Yet, for individuals with mental illness, the large and broadly functional social networks reported as they enter formal mental health care dropped over time, dramatically so compared to general population shifts in the same period [29]. Even online, gamers with more depressive symptoms may seek help by leveraging their networks via online channels [31]. As Boydell et al. [32: 184] note, networked pathways to care appear to be critical to understanding “how services and supports are *received* and *experienced* over time” (original emphasis).

Attention to matters of process, culture, networks, and inequality in health and health care is hardly new in sociomedical science (e.g., [33–36]). How individuals perceive the healthcare systems and its providers, particularly whether they trust physicians, has been a mainstay of understanding service use (e.g., [37–39]). The NEM made no claim of originality. It more intentionally conceptualized formal mental health care utilization as the result of the intersection of community and treatment systems, building a stronger bridge between the “social” and the “psychiatric” [40, 41]. Perhaps no surprise, then, that mental health care utilization researchers who focus on cultural minorities, cultures outside of the West, or other economically and socially disadvantaged populations often used the NEM [42]. For example, among Chinese Americans, conflict in the family network was associated with mental health care utilization [43], but traditional measures of family support were not (also [44, 45] on Korean Americans). Among African Americans, the contrast between kin networks being critical in everyday life but locked out from participation in community mental health centers translated into non-retention in care [46]. Several studies documented how migrants selectively activated “compatriots” (e.g., those with similar experiences), relying on them more heavily as their mental health career progressed and reducing the time to entering formal mental health care [25, 47, 48]. More generally, larger social networks resulted in greater utilization of mental health care among homeless individuals [49], and both youth and older adults [32, 50–52].

However, networks do not always facilitate utilization, as research on the “dark side” of social ties has documented [53]. In some contexts, networks may constrain behaviors and discourage seeking both formal and informal mental health care. For example, among those in Puerto Rico, larger, more supportive networks diminished the uptake of care-seeking patterns that included mental health providers [54] (also [55] on prenatal care). Finally, individuals often employ care providers and practices that never reach the formal mental health care system [56–59]. As Alegria et al. [13] conclude, community, family, friends, and individuals may encourage or block individuals’ referral, entry, or retention in mental health care or substance abuse treatment.

In total, the last 2 decades have compiled a promising and solid body of research that not only offers new insights but provides novel directions for formal and informal mental health care and interventions designed to facilitate pathways to care. Yet, comparatively speaking, little research directly ties community network cultures to individuals’ mental health care-seeking. In some cases (e.g., using statistics for organizational planning), the deep dive into process and communities is not required. However, when the target is to improve entry, retention, and effectiveness, looking to networks continues to be promising. Network data are hard to come by, because research designs that collect such data are resource and time intensive. On the quantitative side, they take more time to collect and require novel analytic tools beyond standard statistical approaches requiring independence of cases [41, 60]. On the qualitative side, efforts to precisely guide qualitative studies are rare and quite recent [61–64]. As Wyke et al. [65: 82–83] conclude, “The idea of a social network as the fundamental unit of analysis is attractive but is easier to articulate than to operationalize.”

Here, we take advantage of the Person-to-Person Health Interview Study (P2P), a representative sample of individuals (*N* ~ 2700) collected between 2018 and 2021 to explore mental health care-seeking pathways. Using P2P data on the structure of respondents’ health-targeted social networks and their trust in the medical system, we examine if and how these factors are related to the number, type, and patterns of mental health care-seeking for past-year problems seen to touch mental health. We draw from the NEM but take an exploratory approach because this stands among the few large-scale, population-representative studies that ask about networks, mental health care-seeking behavior, and mental health care utilization.

Mental health is an important concern in Indiana, where access and utilization of formal mental health care are limited and differential relative to characteristics, such as age, race, gender, rurality, and insurance coverage. A Kaiser Family Foundation fact sheet on mental health in Indiana reports levels of the three most common mental health concerns (anxiety, depression, and mania/hypomania) at levels slightly lower than but not significantly different from those for the United States overall in 2021 (28.6% in Indiana, 31.6% for the US, [66]) That same report indicates that a slightly higher percentage of Indiana residents needing formal mental health care reported not receiving those services relative to levels for the entire US (29.3% v. 26.9%, [66]). Health disparities are a significant problem in Indiana with about a third of Indiana residents reporting common mental health concerns and about a third of those reporting unmet need. Furthermore, the House Committee on Ways and Means reported that in 2020 Indiana had lower access to healthcare, rates of health insurance, average life expectancy, and median household income than the United States overall. Health inequities are more pronounced in areas of Indiana with higher proportions of non-white residents [67, see also 68, 69]. This study explores pathways to mental health care. In light of the clear presence of health disparities based on education, socioeconomic status, insurance status, race, and ethnicity a study that explores such pathways from the point of view of key characteristics associated with health disparities in the United States stands to provide unique information for policy change.

## Methods

### Data source and study sample

The Indiana University Person-to-Person Health Interview Study (P2P) is an omnibus health and wellness study based on face-to-face interviews, designed to study multilevel factors that shape health, using a stratified probability sample of households across the state of Indiana. In addition to demographics, the study collected information on a broad range of health behaviors and attitudes, service utilization and attitudes, employment history, environmental exposure through work and home, and an ego-centered network battery. The study was conducted with a target random sample of 2700 State residents selected to be representative on age, ethnicity, urbanicity, county (a proxy for economic status), and gender. Data were collected from October 23, 2018, to March 21, 2020 when interviewers were pulled from the field as COVID-19 became prevalent in Indiana. Interviews resumed from July 16, 2020 to June 30, 2021 (*N* = 2685). After deletion of respondents with missing age, race, sex, number of total adults in the household, or key mental health outcomes, the effective sample for this analysis is *N* = 2559 individuals.

Respondent enrollment occurred in two stages. First, our sampling design partners, NORC, developed a randomized state-level household sampling strategy to be reflective of the population distribution of the state but clustered within 50% of counties to facilitate data collection. This approach is identical to the approach used in the US General Social Survey, which NORC implements. Then, each household is approached and an adult is asked to describe all adult members of the household. From that list, a survey respondent is chosen at random to participate in the study. If they refuse to participate, the field interviewer moves on to the next randomly selected household on the list. Participation in the study requires only that a respondent be an adult cognitively able to respond to an interviewer administered survey. Thus, these data avoid the limitations of surveys using convenience samples or respondent panels, which are vulnerable to response and selection bias, particularly for those with lower education level or living in rural communities. Our focus here is on individuals who self-reported having a mental health/emotional problem in the past year (*N* = 400). Examining pathways to care from a population point of view requires that individuals or those around them suggest that there may be a problem. Of course, pathways can be examined in treated populations sampled from clinical records with interview follow-up [54] or looking at the service use of individuals with “need” determined by population-based diagnostic measures.^1^

### Measures

#### Mental health problem: severity and use variables

Measures were developed specifically for examining the response to self- or other-perceived mental health problems. Respondents were asked if they thought or someone around them thought that they (respondent) might have a mental health or emotional problem. For participants responding “yes”, follow-up questions included problem severity assessed on a four-point scale ranging from very serious (1), moderately serious (2), not very serious (3) to not serious at all (4). Eight single utilization items asked who they talked to (yes/no) about those problems: relative; friend; neighbor; coworker; minister, pastor, or priest; physician, nurse, physician’s assistant, nurse practitioner or community health worker; psychologist, psychiatrist, social worker or counselor; or nobody.

#### Network variables

Network data were collected using the PhenX Toolkit social network measures (https://www.phenxtoolkit.org/protocols/view/211101). Three variables included network size, a count of people respondents listed as important matters or health ties (range to 20). For each person listed, respondents rated relationship strength (scale 1–10). Average relationship strength served as a general measure of connection. For each, they also indicated how much they felt the ties trusted doctors to take care of people’s problems (responses: none, a little, some, a lot). Pro-medical cultural climate for each respondent was calculated as the percentage of ties who were thought to trust doctors “a lot”. The P2P did not do a second-stage interview of network ties as in some studies (e.g., [54]) because studies have reported that respondents accurately report information on network close or frequent ties [48, 70].

#### Sociodemographic, need, and cultural controls

Network variables are not the only correlates, given the solid body of research on mental health care disparities [13]. Social and cultural characteristics mark important limits on contacts [40, 54: 227]. Respondent age was calculated as the difference between date of interview and birthdate. Participants were asked about their current sex or gender: male, female, and other identities (e.g., transgender, non-binary/gender fluid). Due to the very small sample size of alternative identities, we use the simple male/female dichotomy. Race compares non-Hispanic white participants with all other races and ethnicities due to limited number of respondents, in line with Indiana’s population profile. Education was recorded as the highest level of education completed in five categories: less than high school, high school graduate/GED, some college (no degree), and technical certificates/associates degree, or college and higher. Rural status is based on the county residence determined by NORC’s Metropolitan/Micropolitan status codes. Table 1 provides the basic sample descriptives. In addition, perceived severity variable is included as a continuous variable.

**Table 1:** Demographic data, pathways analysis, Person-to-Person (P2P) Health Interview Study, 2018–2021 (effective sample size 2559)

Age	
Average (std. dev)	39.5 (15.6)
18 to < 35 years	198 (49.5%)
35 to < 50 years	101 (25.3%)
50 to < 65 years	68 (17.0%)
65 years and older	33 (8.3%)
Race	
Minority	53 (13.3%)
White	347 (86.8%)
Gender	
Male	116 (29.0%)
Female	280 (70.0%)
Transgender^a^	4 (1.0%)
Education	
< HS	41 (10.3%)
HS or GED	108 (27.0%)
Some college	106 (26.5%)
Techn/assoc deg	50 (12.5%)
College deg	95 (23.8%)
Rurality	
Urban	284 (71.0%)
Rural	116 (29.0%)

^a^The transgender category presented in this table includes those who identified as transgender, non-binary or gender fluid, genderqueer, intersex, or any other non-cis gender identities

### Statistical analysis

Descriptive statistics reported demographics, mental health problems, and network variables. To identify pathways, latent class analysis used the eight utilization items. Automatic starting values for the Rho parameters were used and set a seed for reproducibility. No covariates were used in the initial pathway analyses. Selection of the final class model was based on model fit criteria, primarily the Bayesian Information criteria (BIC, lower values indicate better fit) and interpretability. Other fit statistics are also reported. To assess pattern correlates, multinomial regression used predicted class membership as the outcome with severity, network structure and culture, and demographic variables (age, gender, race, education, and rural status) as predictors.

## Results

Table 2 presents the basic descriptive data on perceived mental health problems, social networks, and source of care. Of the four hundred individuals reported that they or someone they knew felt they had a mental health or emotional problem, most (77.1%) reported the problem as moderate or very serious. Respondents most frequently reported talking to relatives (74.0%); friends (67.3%); or a physician, physician’s assistant, nurse, nurse practitioner, or community health worker (49.5%) about their mental health or emotional problem. Respondents reported social networks with an average of about six unique members named across name generators (5.7), strong relationships with network members (average tie strength of 8.1 on a 1–10 scale), and that about half of their network members (50.4%) trusted doctors very much.

**Table 2:** Descriptive data, pathways analysis, Person-to-Person (P2P) Health Interview Study, 2018–2021 (effective sample size 2559)

	*N* (%)
Severity of mental health or emotional problem	
Very serious	100 (25.3%)
Moderately serious	205 (51.8%)
Not very serious	71 (17.9%)
Not serious at all	20 (5.1%)
Did you talk with any of the following about this mental health or emotional problem?	
Relative	296 (74.0%)
Friend	269 (67.3%)
Physician, nurse, PA, NP, community health worker	198 (49.5%)
Psychologist, psychiatrist, social worker, or counselor	175 (43.8%)
Coworker	81 (20.3%)
Minister, pastor, or priest	40 (10%)
Neighbor	34 (8.5%)
No, didn’t talk to any type listed	13 (3.3%)
Network size	5.7 (3.0)
Average tie strength	8.1 (1.4)
Average proportion of alters that trust doctors a lot	50.4 (33.6)

### Pathways to care

The latent class analyses reveal five groups, based on patterns of communication related to the reported mental health or emotional problem (Table 3). With data density using a 40% probability of endorsing a care source, the largest pathway group (37%) was a Networked, General Care (NGC) pathway. Almost all these respondents spoke to family (72%) and friends (96%) with nearly half (43%) visiting a general health care provider. The next largest group (32.5%) activated varied and numerous sources of advice and care. In this Networked Multi-Sector Care (NMC) path, respondents spoke to family (62%) and friends (55%) with two-thirds visiting a specialty mental health provider (66% mentioning psychologist, psychiatrist, social worker, or counselor) and nearly half accessing providers in the general care sector (45%). In the Kin General Care (KGC, 14.5%) path, all spoke to relatives (100%) but fewer than half visited a general medical sector (40%). While the Saturated Path (SP) represents only 12.8% of respondents, they activated the most sources of care—family (100%); friends (97%); coworkers (58%); a minister, pastor, or priest (45%); and both general (88%) and specialty sector (92%) providers. The smallest group (3.3%) spoke to no one, representing a Null path (NP).

**Table 3:** Results from LCA—5 group solution, pathways analysis, Person-to-Person (P2P) Health Interview Study, 2018–2021 (effective sample size 2559)

Class	1	2	3	4	5
Gamma estimates (class membership probabilities)	0.340	0.033	0.124	0.343	0.162
People sometimes talk to others about their problems. Did you talk with any of the following about this problem?	The Networked General Care Path	The Null Path	The Kin-General Care Path	The Networked Multi-sector Care Path	The Saturated Path
Response category YES	Rho estimates (item response probabilities)				
Relative	0.72	0	1	0.62	1
Friend	0.96	0	0	0.55	0.97
Neighbor	0.10	0	0	0.02	0.28
Coworker	0.32	0	0.00	0	0.58
Minister, pastor, or priest	0.06	0	0.07	0	0.45
Psychologist, psychiatrist, social worker, or counselor	0.15	0	0.10	0.66	0.92
Physician, nurse, physician’s assistant, nurse practitioner or community health worker	0.43	0	0.40	0.46	0.88
No, didn’t talk to anyone listed	0	1	0	0	0
Frequency	148	13	58	130	51
Percent	37.0%	3.3%	14.5%	32.5%	12.8%

### Pathway correlates

The multinomial regression results reveal the relationship between pathways, network and need factors, and demographic controls (Table 4). Wald Chi-square tests provide an assessment of whether each correlate has an overall effect on pathway type. Severity of the problem and network size significantly discriminated across care pathways. Age and race were marginally associated.

**Table 4:** Multinomial logistic regression main effects (with controls and networked general care path as reference category), pathways analysis, Person-to-Person (P2P) Health Interview Study, 2018–2021 (effective sample size 2559)

Type 3 analysis of effects			
Effect	*df*	Wald Chi-square	Pr > ChiSq
Problem severity	4	29.6328	< 0.0001
Network size	4	14.5588	0.0057
Average tie strength	4	4.6276	0.3277
Network trust in doctors	4	7.3118	0.1203
Age	4	8.8865	0.0640
Gender	4	4.3521	0.3605
Education	12	15.3120	0.2248
Race	4	8.1620	0.0858
Rurality	4	5.8511	0.2105

**Fig. 1:**
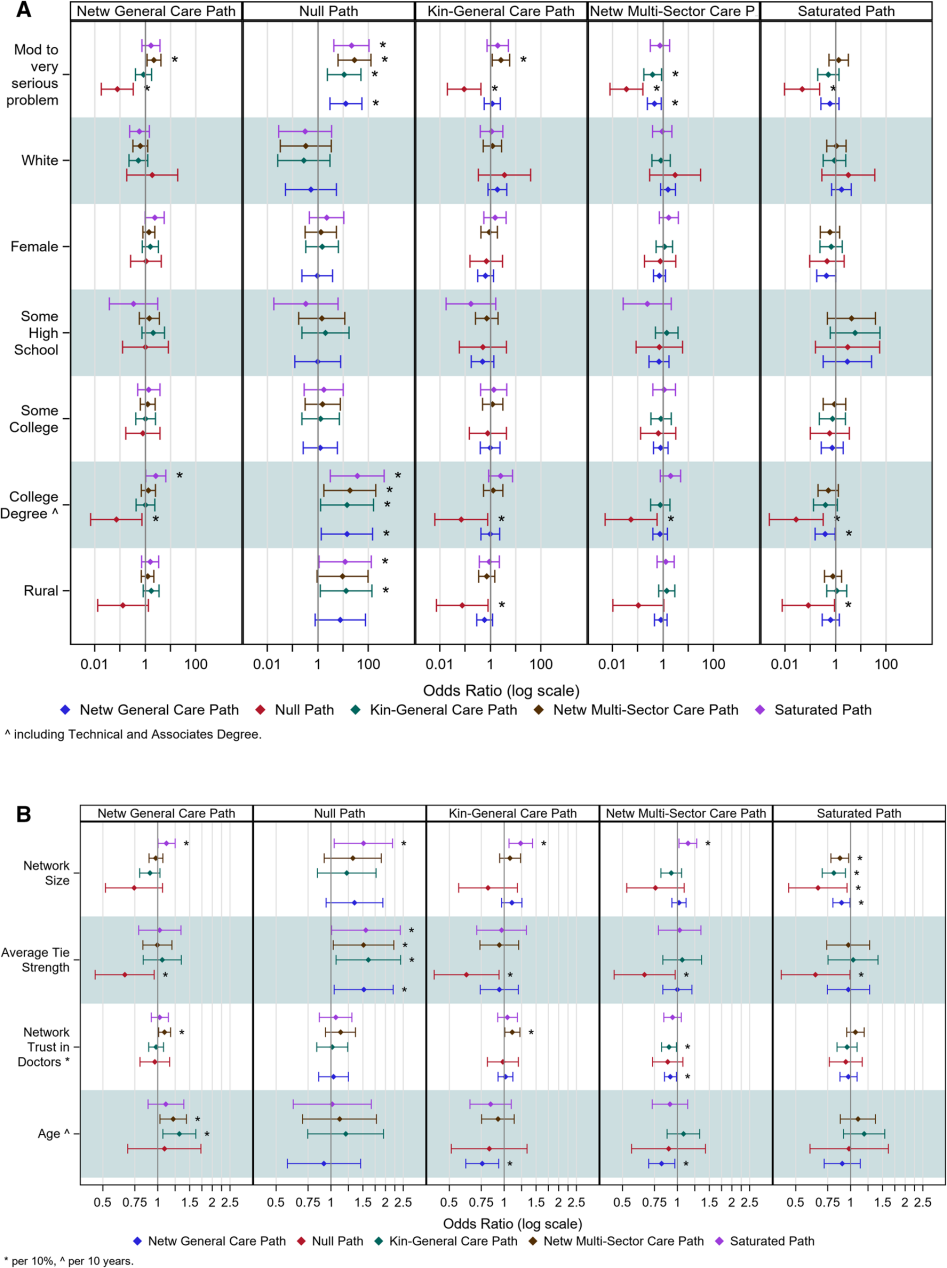
**a** Odds ratios and standard errors for multinomial regression models across all reference categories, continuous and ordinal variables. Relevant reference category is indicated by color. Significant effects are noted with an asterisk. Person-to-Person (P2P) Health Interview Survey, 2018–2021 (effective sample size 2559). **b** Odds ratios and standard errors for multinomial regression models across all reference categories, binary variables. Relevant reference category is indicated by color. Significant effects are noted with an asterisk. Person-to-Person (P2P) Health Interview Study, 2018–2021 (effective sample size 2559)

A more fine-grained analysis requires the sets of multinomial regressions where correlates are examined on each contrast of two pathways. Figure 1a, b provides a graphical summary of effects from the eight multivariate tables (one full contrast provided in Appendix 1, full set on request). These analyses, more detailed and relatively more complicated than typical regression results, are interpreted as follows: each column represents a reference category in a multivariate regression that examines the impact of independent variables on whether individuals were more likely to travel a different pathway compared to the reference. Each table row provides the effects of an independent variable on that comparison for a particular comparison group, indicated by a color-coded whisker plot. It should be noted that the whisker plots associated with the reference category are not included. For example, the upper left corner of Fig. 1a represents the four analyses done to see if and how network size affects whether individuals activated an alternate pathway compared to the Networked General Care Pathway (NGC), with the blue whisker plot corresponding to NGC not shown. The one, starred effect indicates a significant effect of network size—those with larger networks are more likely to report the purple path (SP) compared to the reference NGC. Substantively, the interpretation suggests that those with larger networks are likely to go beyond activating family and physician helpers (in the NGC), because the saturated path includes coworkers, friends, the clergy, and specialty providers. Looking across all cells in that top row indicates that larger networks make the saturated pathway the most common as network size increases. In turn, this indicates that when human resources are available, individuals will activate them and social networks can facilitate help-seeking across the board.

The network strength findings (next set of rows) indicate that most of the action is located relative to the Null Path. This indicates that, controlling for network size, those who have stronger ties are more likely to travel any pathway rather than not do anything at all. Trust in doctors does not have many significant effects; however, trust predisposes individuals to paths that include the specialty sector (see columns 1, 3) but does not generally increase types of care (non-significance in column 5). Older respondents were more likely to report pathways that included relatives alone (columns 1,3), but when they do include friends, they are more likely to include specialty care (columns 1,4).

In Fig. 1b, individuals who reported their problem to be moderate or serious were more likely to travel any path than the Null Path (column 2); however, they were also more likely to end up in pathways that included specialty providers (columns 1, 3). Whites were more likely to travel pathways that included specialty providers while individuals from minority populations were less likely to do so (column 1). Gender had no effect on pathways. The effects of education come into play only for those with college/advanced degrees. In this case, respondents travel any pathway rather than do nothing and are less likely to stop at the general care sector. Rurality has inconsistent effects but suggests that rural residents are more likely to do nothing than engage either a lot of others or, if they do get formal care, tend to activate only kin and general medical care (column 5).

## Discussion

Our ability to understand the role that “the social” plays in mental illness is undermined, at least in part, by a reliance on a view that focuses on the individual alone. While sociodemographic categories tap into the social and cultural lives of individuals, providing critical insights, they are less well equipped to see how those factors operate to create inequalities. Research in many areas, including employment, migration and immigration, and birth control, has established that human connections are often those active ingredients creating disparities, even for health behavior change [71]. Network research, which traces the human connections that surround individuals and stand as the sources of formal and informal care than can be activated during a health crisis, may offer novel findings. Yet, because network data collection is messy, complicated, and more time-consuming than the traditional quantitative or qualitative approaches [60], social network studies are more rare. However, we argue that the Network Episode Model may be a better theoretical match, because the search for care, especially for mental health problems, also appears to be messy and complicated.

The findings reported here are far from meeting the immensity of that challenge. However, we attempted to contextualize the search for mental health care by connecting individuals to whom they consulted about their problem and enumerating the structure and cultural content of those network ties. In this representative sample, we find that almost 15% of individuals reported problem recognition by self or other and their response pathways varied from doing nothing (a very small number) to activating lay and professional ties across many sectors of the community, including family, friends, coworkers, and general and specialty medical providers. Both networks and severity propel individuals into care-seeking behavior. While those with strong ties are likely to seek care of some sort, having larger networks translates into accessing a richer set of options. Cultural beliefs in those networks matter to some extent, since networks with strong trust in doctors are associated with pathways that access the general and specialty medical sectors. Individuals from the majority white population, and those with higher levels are education, report pathways to the specialty sector, signaling a continued disparity.

Of course, this study is not without problems that raise questions about disparities. In our representative sample, we find few race effects. While it may be unsurprising that minority population respondents are less likely to report pathways that include psychiatric care providers than are majority individuals, there are both sampling and prevalence limits here. First, even with a population-based sample of nearly 3000 individuals, we had only a small group that reported self- or other-perceived need, as indicated above. Second, this is compounded by fielding the study in a state that has about 10% black population and another 6% other races and/or ethnicities. Only an oversample would offer a more solid basis for examining race and ethnic disparities and the social processes involved in the search for mental health care. However, in a state that is highly rural 78% of counties, and where education ranks 46th nationally in adults with a college degree, we find both residence and education in operation to some extent. Finally, the stem of the mental health/emotional problem question does not distinguish between common or severe mental health problems or distinguish between individuals who were self-aware or who were nudged, or even coerced, by others to recognize and act on their mental health issues. We know that pathways, and network effects on pathways, are shaped by whether coercion or agency is at work in seeking care [22, 54]. Finally, as noted earlier, we have data from respondents on their social network members. We cannot ascertain whether respondents’ self-reports are accurate interpretations of the beliefs and opinions of their network ties. Ultimately, the NEM may be best suited to a mixed method approach or to one that includes oversamples of minority populations [72]. To that end, a mixed-methods study involving qualitative interviews exploring pathways to care, particularly among minority populations is underway and subsequent survey administrations will feature oversamples of minority populations. Subsequent studies will also disaggregate respondents according to assessments of depression, anxiety, and mania/hypomania.

Nevertheless, our goal here was to reconsider the role of “the social” in understanding the lay and professional resources that individuals use over the course of seeking care for a mental health problem by looking to the structure and culture of their social networks. We have established that there are unique pathways to mental health care. Further, social networks and the nature of the problem matter most in the response to mental health problems. Having stronger ties translates into eliciting a response, but only having many ties and ones that support medical solutions result in activating a lot of helpers, including those in the specialty mental health sector. As we see social ties exert influence, it becomes imperative to think about utilization as a community response, even if we think of “small worlds” within communities. In turn, policy and services designed to alleviate the burden of mental health problems might be more effective if they shift from a focus on individuals to a focus on their communities.

## Data Availability

The data that support the findings of this study are not publicly available at the time of publication. The data are, however, available from the authors upon reasonable request and with the permission of the Person-to-Person Health Interview Study Principal Investigators. To inquire about access to P2P data, please email irsay@iu.edu.

## Appendix 1

See Table

**Table 5:** Multinomial logistic regression main effects (with controls and networked general care path as reference category), pathways analysis, Person-to-Person (P2P) Health Interview Study, 2018–2021 (effective sample size 2559)

Effect	The networked general care path (NGC)	The null path (NP)	The kin-general care path (KGC)	The networked multi-sector care path (NMC)	The saturated path (SP)
Problem severity	Reference	5.33* (2.03, 13.99) ***	1.00 (0.66, 1.50)	0.56 (0.40, 0.79) ***	0.53 (0.33, 0.84) **
Network size	Reference	0.67* (0.47, 0.97)	0.91 (0.80, 1.04)	1.00 (0.91, 1.09)	1.15 (1.03, 1.28) *
Average tie strength	Reference	0.68* (0.46, 0.99)	1.06 (0.83, 1.35)	0.98 (0.81, 1.18)	1.01 (0.77, 1.32)
Network trust in doctors	Reference	1.00 (0.98, 1.02)	1.00 (0.99, 1.01)	1.01 (1.00, 1.02) *	1.00 (0.99, 1.01)
Age	Reference	1.01 (0.97, 1.06)	1.03 (1.01, 1.05) **	1.02 (1.00, 1.04) *	1.01 (0.99, 1.03)
Gender Ref = male	Reference	1.40 (0.34, 5.71)	1.52 (0.73, 3.18)	1.37 (0.79, 2.37)	2.31 (0.97, 5.51)
Education some high school	Reference	1.07 (0.13, 8.59)	1.98 (0.7, 5.59)	1.45 (0.58, 3.63)	0.34 (0.04, 3.11)
Some college	Reference	1.02 (0.21, 4.98)	1.01 (0.41, 2.49)	1.19 (0.60, 2.36)	1.29 (0.46, 3.58)
Technical, assoc, college degree Ref = GED or HS diploma	Reference	0.09* (0.01, 1.00)	1.03 (0.44, 2.44)	1.30 (0.67, 2.51)	2.62 (1.05, 6.57) *
Race Ref = minority	Reference	0.55 (0.05, 6.16)	2.58 (0.98, 6.84)	2.65 (1.19, 5.89) *	2.91 (1.03, 8.18) *
Rural Ref = urban	Reference	6.66 (0.66, 67.31)	0.58 (0.29, 1.20)	0.81 (0.46, 1.43)	0.64 (0.29, 1.39)

* indicates a *p*-value < 0.05, ** indicates a *p*-value < 0.01, and *** indicates a *p*-value < 0.001

^5^.

## Footnotes

1 Instrumentation varies with the study population. For those in care, the pathway stem question would be: How did you get here? For population-based samples like the one here, the stem question is the problem recognition question listed in the text.
